# Reassessing prognostic markers in metastatic renal cell carcinoma in the era of immune checkpoint inhibitors: the enduring value of body composition, nutritional, and inflammatory indices

**DOI:** 10.1007/s10147-025-02855-6

**Published:** 2026-01-23

**Authors:** Norihiko Tsuchiya, Sei Naito, Hiroki Fukuhara, Hayato Nishida, Mayu Yagi, Yuki Takai, Atsushi Yamagishi, Takafumi Narisawa, Shinta Suenaga

**Affiliations:** Faculty of Medicine, Department of Urology, Yamagata University, 2-2-2 Iida-Nishi, Yamagata, 990-9585 Japan

**Keywords:** Metastatic renal cell carcinoma, Immune checkpoint inhibitor, Body composition, Nutritional status, Systemic inflammation

## Abstract

**Background:**

Immune checkpoint inhibitors (ICIs) are now the standard first-line treatment for metastatic renal cell carcinoma (mRCC), yet many risk factors identified during the tyrosine kinase inhibitor era remain unvalidated in current practice. This study aimed to evaluate the prognostic value of body composition, nutritional, and inflammatory indices in the era of ICI-based first-line therapy.

**Methods:**

We retrospectively analyzed 136 mRCC patients who received systemic therapy. Body composition indices (skeletal muscle index [SMI], visceral adipose tissue index [VATI], subcutaneous adipose tissue index [SATI]), nutritional markers (prognostic nutritional index [PNI], geriatric nutritional risk index [GNRI]), and inflammatory markers (Glasgow Prognostic Score [GPS], systemic inflammatory index [SII], and other indices) were assessed for their association with overall survival (OS). We also compared their prognostic impact on patients treated with non-ICI-based and ICI-based regimens as first-line therapy.

**Results:**

Low VATI (HR 1.64, *P* = 0.030), and low SATI (HR 2.22, *P* < 0.001) were associated with shorter survival. PNI (HR 1.70, *P* < 0.001) and GNRI (HR 1.57, *P* < 0.001) showed strong prognostic value, as did GPS (HR 2.43, *P* < 0.001) and SII (HR 2.11, *P* < 0.001) in the overall cohort. In the ICI-based regimen group, PNI, GNRI, and SATI demonstrated higher prognostic performance (C-indices 0.736, 0.730, and 0.690, respectively), with PNI and SATI providing clear OS stratification.

**Conclusion:**

Several indices reflecting body composition, nutritional status, and systemic inflammation remain valuable prognostic markers in patients with mRCC receiving ICI-based first-line therapy.

**Supplementary Information:**

The online version contains supplementary material available at 10.1007/s10147-025-02855-6.

## Introduction

First-line systemic therapy for advanced renal cell carcinoma (RCC) or metastatic RCC (mRCC) has increasingly adopted combination strategies using tyrosine kinase inhibitors (TKIs) and immune checkpoint inhibitors (ICIs), which have led to substantial improvements in patient prognosis [1]. The ICI-based therapy has markedly prolonged survival, highlighting the need for a new risk stratification system beyond the International Metastatic RCC Database Consortium (IMDC) risk stratification, which was originally developed based on outcomes from anti-vascular endothelial growth factor-targeted therapies [2]. Given the wide range of available treatments for mRCC, more accurate risk classification is essential to select the optimal therapy. While conventional prognostic models are based on clinical and hematological/biochemical parameters and offer practical utility, recent advances in systemic therapy underscore the potential value of incorporating prognostic factors from alternative domains. Such factors may contribute to improved prognostic accuracy in the current therapeutic landscape.

Previous epidemiological studies have reported that obesity is a risk factor for RCC [3], while obesity or increased adipose tissue mass has been reported as a favorable prognostic factor for patients with RCC [4, 5]. Obesity being a risk factor for diseases while also being a favorable prognostic factor is known as the “obesity paradox” [6, 7]. Although this paradox remains unexplained, many hypotheses have been put forward. The mechanisms by which obesity affects disease prognosis have been suggested as reverse causation and possibly confounded by other prognostic factors and treatment effect determinants rather than a direct effect of obesity [6].

Various indicators that represent the physical state of the patient, such as nutritional status and inflammation, have been reported to be possible predictors of cancer prognosis [8, 9]. The prognostic nutritional index (PNI) was initially developed as an indicator of nutritional status to assess the risk of surgical complications of gastrointestinal malignancies [10], and the geriatric nutritional risk index (GNRI) was developed to assess nutritional risk in the elderly [11]. Complete blood count–derived parameters, such as the neutrophil-to-lymphocyte ratio (NLR) and platelet-to-lymphocyte ratio (PLR), are indicators that reflect silent inflammation [12]. The Glasgow Prognostic Score (GPS) is a unique prognostic indicator that reflects both nutritional status and systemic inflammation, based on serum albumin levels and C-reactive protein concentrations [13]. These nutritional and inflammatory indices indicate the prognosis of RCC [12–14].

Most of these markers, however, were identified in the TKI era, and their prognostic significance in the era of ICI-based therapy is yet to be established. Therefore, this study aimed to evaluate the prognostic significance of body composition, nutritional, and inflammatory indices in patients with mRCC treated with ICI-based first-line regimens and to compare their predictive performance to identify those with superior prognostic utility.

## Materials and methods

### Patient selection and ethical considerations

A total of 202 patients with mRCC received first-line systemic therapy from 2010 to 2023 at Yamagata University Hospital in Japan. A total of 136 patients who received TKIs, mammalian target of rapamycin inhibitors (mTORIs), ICI + ICI, or ICI + TKI as first-line therapy and for whom evaluable CT imaging and relevant clinical data were available for assessing body composition, nutritional status, and inflammatory markers were included in the analysis. Thirty-two patients were not included in the analysis as they received first-line therapies other than TKI- or ICI-based regimens, 14 were excluded because of the unavailability of CT imaging, 20 were omitted because of missing biochemical or hematological data (Supplementary Fig. S1). This study was approved by the institutional review board of the Faculty of Medicine at Yamagata University (2018-43). An opt-out method was used to obtain informed consent.

### Measurement of muscle and adipose tissue components

Patients’ clinical and CT imaging data were obtained immediately before the first-line treatment. The measurement of muscle and adipose tissue components was conducted using the Automated Muscle and Adipose Tissue Composition Analysis (AutoMATiCA) system [15]. This open-source software was obtained from https://gitlab.com/Michael_Paris/AutoMATiCA. Subcutaneous adipose tissue area, visceral adipose tissue area, and skeletal muscle area were automatically measured at the L3 level. The skeletal muscle index (SMI), visceral adipose tissue index (VATI), and subcutaneous adipose tissue index (SATI) were defined as the corresponding area divided by body height squared. The visceral-to-subcutaneous adipose tissue ratio (VSR) was calculated as the visceral adipose tissue area divided by the subcutaneous adipose tissue area. The skeletal muscle index (SMI) was used as an index of sarcopenia and classified based on the criteria by Martin et al. [16] Briefly, patients were categorized into the sarcopenia group if they had body mass index (BMI) < 25 with SMI < 43 cm^2^/m^2^ or BMI ≥ 25 with SMI < 53 cm^2^/m^2^ for males, and SMI < 41 cm^2^/m^2^ regardless of BMI for females. No universal cutoff values exist for VATI, SATI, or VSR as cancer prognostic markers. Optimal thresholds were determined by receiver operating characteristic (ROC) analysis using the Youden index, with cutoff values set at 18.6 cm^2^/m^2^ for VATI, 30.0 cm^2^/m^2^ for SATI, and 1.6 for VSR. A BMI of < 18.5 kg/m^2^ was defined as underweight, 18.5–25.0 kg/m^2^ as normal weight, ≥ 25.0 kg/m^2^ as overweight, and ≥ 30.0 kg/m^2^ as obese.

### Calculation of nutritional and inflammatory indices

The PNI was calculated using the following formula: 10 × serum albumin (g/dL) + 0.005 × total lymphocyte count (/µl) [10]. On the basis of the PNI score, nutritional status was classified into three categories: no malnutrition: PNI ≥ 45; mild malnutrition: 40 ≤ PNI < 45; and malnutrition: PNI < 40 [17]. The formula for GNRI is [14.89 × serum albumin (g/dL)] + [41.7 × (weight/WLo)], where WLo is an ideal weight calculated from the Lorentz equations [11]. GNRI was categorized as follows: ≥ 98: no risk; ≥ 92 and < 98: low risk; ≥ 82 and < 92: moderate risk; and < 82: major risk [11]. The GPS was used as a marker of both nutritional status and systemic inflammation [18] and is a composite index ranging from 0 to 2, with one point assigned for a C-reactive protein level of > 1 mg/dL and one point for a serum albumin level of < 3.5 g/dL. The systemic immune inflammation index (SII), neutrophil-to-lymphocyte ratio (NLR), platelet-to-lymphocyte ratio (PLR), and lymphocyte-to-monocyte ratio (LMR) were used as systemic immune-inflammatory biomarkers. SII was calculated from the equation: P x N/L, where P, N, and L are the pretreatment peripheral blood platelet, neutrophil, and lymphocyte counts, respectively [19]. SII was dichotomized using a cut-off value of 788 × 10^9^/L, based on a previous study [20]. Similarly, the cut-offs for NLR, PLR, and LMR were set as 3.0 [20], 150.0 [21], and 3.0 [22], respectively, based on previous reports and meta-analyses.

### Statistical analysis

R software version 4.4.2 (R Foundation for Statistical Computing, Vienna, Austria) was used for statistical analyses. The Mann–Whitney test was used to compare continuous variables between two groups. The Chi-square test was used to compare categorical variables. Overall survival (OS) was defined as the time from initiation of the first-line systemic therapy to death from any cause and was analyzed using Kaplan–Meier curves with a log-rank test, while age and sex adjusted Cox regression analysis was used to assess the effects of each variable on survival. A Pearson correlation coefficient was calculated for each pair of indices. The concordance index (Harrell’s C-index) was calculated to assess the model’s time-dependent discriminatory ability. A *P*-value of < 0.05 was considered statistically significant.

## Results

### Patients’ clinical demographics

The patients’ demographics are summarized in Table 1. A total of 136 patients (108 male and 28 female) were included in the analysis. The median age was 66 years (interquartile range [IQR], 61–71), and the median follow-up duration was 22.7 months (IQR, 8.9–47.2). Pathological confirmation revealed clear cell RCC in 106 patients (78%), followed by papillary RCC (6.6%), chromophobe RCC (3.7%), collecting duct carcinoma (2.9%), acquired cystic disease-associated RCC (2.2%), other subtypes (3.7%), and unknown (2.9%). According to the IMDC risk classification, 8.8% of patients were categorized as favorable risk, 52% as intermediate risk, and 39% as poor risk. The most common first-line regimen was TKI in 57% of patients, followed by ICI-based regimens (ICI + ICI: 29%; ICI + TKI: 9.6%) and mTORI (4.4%).

**Table 1:** Baseline demographics and clinical characteristics of patients according to first-line regimen

Variable	Overall (*n* = 136)	Non-ICI-based regimen (*n* = 84)	ICI-based regimen (*n* = 52)
Age, year (IQR)	66 (61, 71)	68 (63, 71)	68 (63, 72)
*Sex* (%)			
Male	108 (79)	66 (79)	42 (81)
Female	28 (21)	18 (21)	10 (19)
Follow-up, month (IQR)	22.7 (8.9, 47.2)	21.6 (8.8, 43.6)	25.2 (10.5, 49.2)
*Pathological type* (%)			
Clear cell RCC	106 (78)	68 (81)	38 (73)
Papillary RCC	9 (6.6)	6 (7.1)	3 (5.8)
Chromophobe cell renal carcinoma	5 (3.7)	3 (3.6)	2 (3.8)
Collecting duct carcinoma	4 (2.9)	1 (1.2)	3 (5.8)
Acquired cystic disease-associated RCC	3 (2.2)	1 (1.2)	2 (3.8)
Other types	5 (3.7)	2 (2.4)	3 (5.8)
Unknown	4 (2.9)	3 (3.6)	1 (1.9)
*IMDC risk criteria* (%)			
Favorable	12 (8.8)	7 (8.3)	5 (9.6)
Intermediate	71 (52)	49 (58)	22 (42)
Poor	53 (39)	28 (33)	25 (48)
*First-line treatment* (%)			
TKI	78 (57)	78 (93)	‒
mTORI	6 (4.4)	6 (7.1)	‒
ICI + ICI	39 (29)	‒	39 (75)
ICI + TKI	13 (9.6)	‒	13 (25)

*IQR* interquartile range, *RCC* Renal cell carcinoma, *IMDC* International mRCC Database Consortium, *TKI* Tyrosine kinase inhibitor, *mTORI* mTOR inhibitor, *ICI* Immune checkpoint inhibitor

### Sex differences in prognostic markers

Significant sex differences were observed in several body composition indices: males had higher values than females for SMI (48.7 vs. 37.1 cm^2^/m^2^, *P* < 0.001), VATI (33.4 vs. 12.4 cm^2^/m^2^, *P* < 0.001), and VSR (0.9 vs. 0.4, *P* < 0.001). By contrast, SATI did not differ significantly between sexes (32.2 vs. 35.5 cm^2^/m^2^, *P* = 0.761). Among inflammatory markers, PLR was significantly higher in female individuals (261.5 vs. 206.4, *P* = 0.031), while other indices showed no significant sex-based differences (Supplementary Table S1).

### Influence of prognostic markers on OS in the overall cohort

As shown in Table 2, in the overall cohort, low VATI (HR 1.64, 95% CI 1.05–2.56, *P* = 0.030), and low SATI (HR 2.22, 95% CI 1.47–3.34, *P* < 0.001) were significantly associated with shorter OS, whereas BMI, SMI (sarcopenia) and VSR had no significant effect on survival. Nutritional markers demonstrated strong prognostic value: patients with malnutrition, as defined by PNI, had significantly shorter OS (HR 1.70, 95% CI 1.33–2.17, *P* < 0.001), and those at higher risk according to GNRI also exhibited poorer survival (HR 1.57, 95% CI 1.30–1.88, *P* < 0.001).

**Table 2:** Association of indices with overall survival adjusted for age and sex

Variable	Overall			Non-ICI-based regimen			ICI-based regimen		
	HR	95% CI	*P*	HR	95% CI	*P*	HR	95% CI	*P*
IMDC (Poor vs. Intermediate vs. Favorable)	2.25	1.56, 3.24	< 0.001	2.61	1.70, 4.00	< 0.001	2.75	1.17, 6.47	0.021
BMI (Underweight vs. Normal vs. Overweight/Obese)	1.42	0.97, 2.08	0.070	1.39	0.91, 2.14	0.132	1.29	0.60, 2.76	0.510
SMI (Sarcopenia vs. No sarcopenia)	1.28	0.85, 1.93	0.228	1.23	0.77, 1.95	0.388	1.67	0.70, 3.99	0.246
VATI (Low vs. High, cutoff 18.6 cm^2^/m^2^)	1.64	1.05, 2.56	0.030	1.90	1.14, 3.16	0.014	2.11	0.82, 5.48	0.123
SATI (Low vs. High, cutoff 30.0 cm^2^/m^2^)	2.22	1.47, 3.34	< 0.001	1.81	1.13, 2.89	0.014	4.61	1.86, 11.4	< 0.001
VSR (Low vs. High, cutoff 1.6)	1.19	0.73, 1.94	0.489	1.76	1.01, 3.08	0.047	0.89	0.33, 2.37	0.812
PNI (Malnutrition vs. Mild malnutrition vs. Normal)	1.70	1.33, 2.17	< 0.001	1.74	1.31, 2.33	< 0.001	2.70	1.44, 5.04	0.002
GNRI (Major vs. Moderate vs. Low vs. No risk)	1.57	1.30, 1.88	< 0.001	1.74	1.38, 2.20	< 0.001	2.01	1.30, 3.09	0.002
GPS (2 vs. 1 vs. 0)	2.43	1.58, 3.76	< 0.001	2.14	1.31, 3.51	0.003	4.27	1.48, 12.3	0.007
SII (High vs. Low, cutoff 788 × 10^9^/L)	2.11	1.37, 3.24	< 0.001	2.03	1.24, 3.33	0.005	2.63	1.02, 6.83	0.046
NLR (High vs. Low, cutoff 3.0)	2.02	1.31, 3.11	0.002	1.69	1.04, 2.74	0.033	4.19	1.39, 12.6	0.011
PLR (High vs. Low, cutoff 150.0)	1.61	1.05, 2.47	0.028	1.63	1.01, 2.62	0.044	2.25	0.87, 5.85	0.095
LMR (Low vs. High, cutoff 3.0)	1.75	1.16, 2.63	0.008	1.41	0.87, 2.28	0.166	3.59	1.50, 8.57	0.004

*ICI* immune checkpoint inhibitor, *IMDC* International mRCC Database Consortium, *BMI* body mass index, *SMI* skeletal muscle index, *VATI* visceral adipose tissue index, *SATI* subcutaneous adipose tissue index, *VSR* visceral-to-subcutaneous adipose tissue ratio, *PNI* prognostic nutritional index, *GNRI* geriatric nutritional risk index, *GPS* Glasgow prognostic score, *SII* systemic immune inflammation index, *NLR* neutrophil to lymphocyte ratio, *PLR* platelet to lymphocyte ratio, *LMR* lymphocyte to monocyte ratio

GPS emerged as a robust predictor, with higher scores correlating with worse outcomes (HR 2.43, 95% CI 1.58–3.76, *P* < 0.001). Among inflammatory markers, elevated SII (HR 2.11, 95% CI 1.37–3.24, *P* < 0.001), NLR (HR 2.02, 95% CI 1.31–3.11, *P* = 0.002), and PLR (HR 1.61, 95% CI 1.05–2.47, *P* = 0.028), as well as lower LMR (HR 1.75, 95% CI 1.16–2.63, *P* = 0.008), were all significantly associated with shorter OS.

### Comparison of prognostic markers between non-ICI and ICI-based regimens

**Fig. 1:**
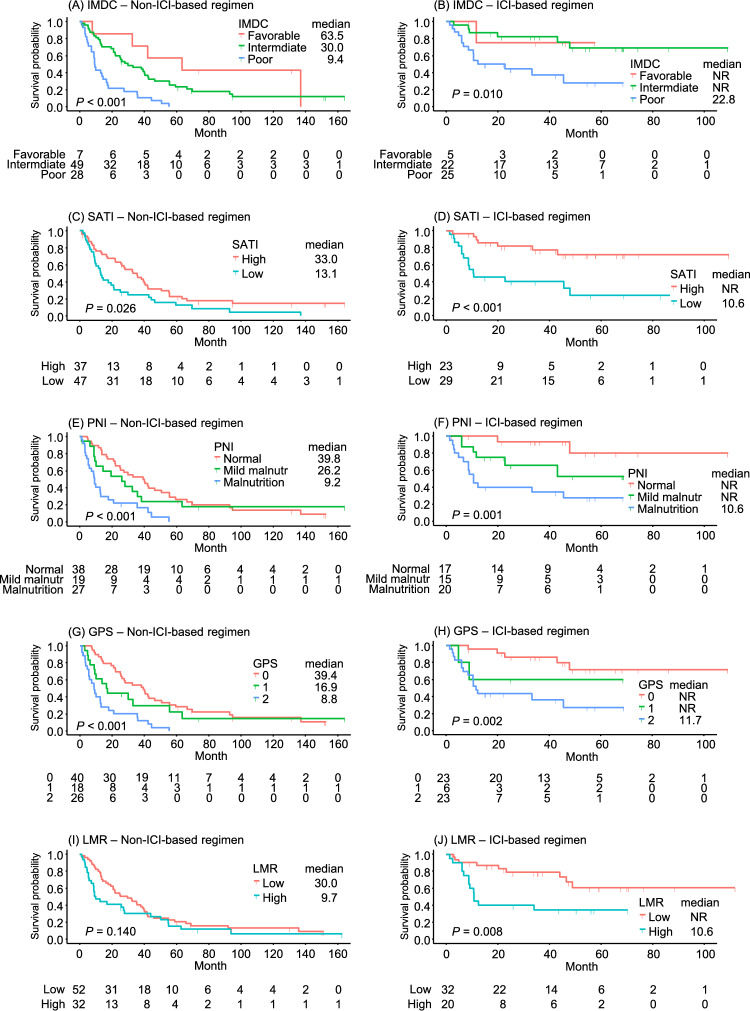
Kaplan–Meier curves of overall survival stratified by IMDC risk classification, SATI, PNI, GPS, and LMR in non-ICI and ICI-based first-line therapy. **A** and **B** IMDC risk classification. **C** and **D** Subcutaneous adipose tissue index (SATI). **E** and **F** Prognostic nutritional index (PNI). **G** and **H** Glasgow Prognostic Score (GPS). **I** and **J** Lymphocyte-to-monocyte ratio (LMR). Left panels (**A**, **C**, **E**, **G**, and **I**) show non-ICI-based regimens; right panels (**B**, **D**, **F**, **H**, and **J**) show ICI-based regimens. The survival probability is plotted over time (months), with log-rank *P*-values and numbers at risk indicated. Median survival times are presented in months for each group, with NR indicating that the median was not reached

There was no significant difference in prognostic markers between the treatment groups at baseline (Supplementary Table S2). Detailed subgroup analyses, as summarized in Table 2, reveal distinct patterns. Kaplan–Meier curves for five prognostic indices, including the IMDC risk classification and four representative indices of body composition, nutritional status, and systemic inflammation, are displayed separately for the two treatment subgroups in Fig. 1. In the non-ICI-based regimen group, low VATI (HR 1.90, 95% CI 1.14–3.16, *P* = 0.014), low SATI (HR 1.81, 95% CI 1.13–2.89, *P* = 0.014), and low VSR (HR 1.76, 95% CI 1.01–3.08, *P* = 0.047) were significantly associated with poorer OS. Nutritional markers, including PNI (HR 1.74, 95% CI 1.31–2.33, *P* < 0.001) and GNRI (HR 1.74, 95% CI 1.38–2.20, *P* < 0.001), also showed strong associations with OS. Inflammatory markers SII (HR 2.03, 95% CI 1.24–3.33, *P* = 0.005), NLR (HR 1.69, 95% CI 1.04–2.74, *P* = 0.033), and PLR (HR 1.63, 95% CI 1.01–2.62, *P* = 0.044), as well as GPS (HR 2.14 95% CI 1.31–3.51, *P* = 0.003), demonstrated significant prognostic value in this group.

Conversely, among patients treated with ICI-based regimens, while the IMDC classification remained significant (HR 2.75, 95% CI 1.17–6.47, *P* = 0.021), Kaplan–Meier curves showed no clear separation between the favorable- and intermediate-risk groups (Fig. 1B). Among body composition indices, SATI emerged as a strong predictor (HR 4.61, 95% CI 1.86–11.4, *P* < 0.001), showing the highest C-index (0.690 as an ordinal variable) among body composition indices (Supplementary Table S3). Both PNI (HR 2.70, 95% CI 1.44–5.04, *P* = 0.002) and GNRI (HR 2.01, 95% CI 1.30–3.09, *P* = 0.002) were significant prognostic factors, demonstrating high predictive performance (C-indices 0.736 and 0.730 as ordinal variables, respectively) (Supplementary Table S3). Notably, PNI showed clear separation of survival curves among the three groups in Kaplan–Meier analysis (Fig. 1F). Among inflammatory markers, GPS (HR 4.27, 95% CI 1.48–12.3, *P* = 0.007), NLR (HR 4.19, 95% CI 1.39–12.6, *P* = 0.011), and LMR (HR 3.59, 95% CI 1.50–8.57, *P* = 0.004) exhibited higher hazard ratios compared with the non-ICI-based regimen group, with survival curves demonstrating significant stratification (*P* = 0.002 for GPS and 0.008 for LMR, respectively) (Fig. 1H and J).

### Correlation analysis among prognostic markers

**Fig. 2:**
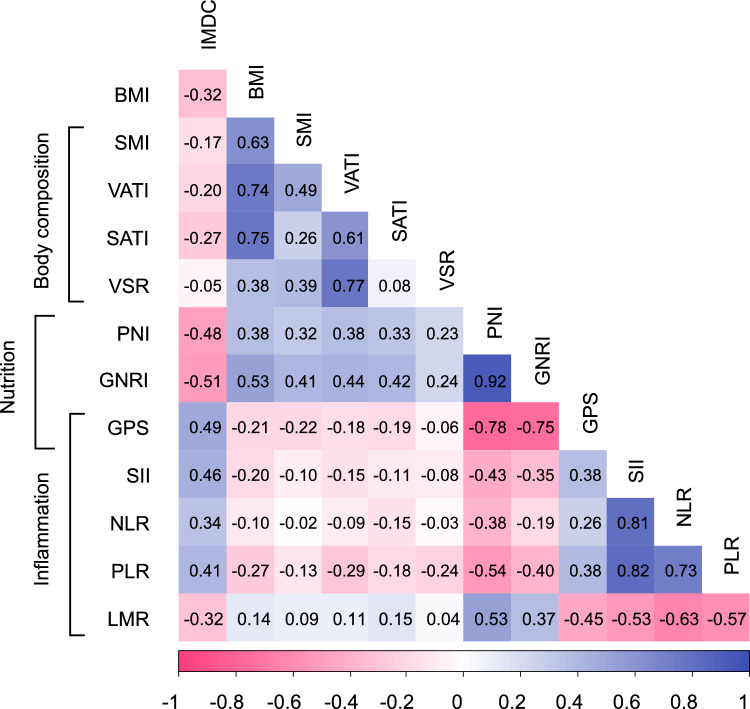
Correlation matrix among body composition, nutritional, and inflammatory indices. The heatmap shows pairwise correlation coefficients between variables including body mass index (BMI), skeletal muscle index (SMI), visceral adipose tissue index (VATI), subcutaneous adipose tissue index (SATI), visceral-to-subcutaneous adipose tissue ratio (VSR), prognostic nutritional index (PNI), geriatric nutritional risk index (GNRI), Glasgow Prognostic Score (GPS), systemic immune inflammation index (SII), neutrophil-to-lymphocyte ratio (NLR), platelet-to-lymphocyte ratio (PLR), and lymphocyte-to-monocyte ratio (LMR). Positive correlations are shown in blue and negative correlations in pink, with stronger correlations indicated by more intense colors

A correlation heatmap of the evaluated indices is shown in Fig. 2. Moderate correlations were observed between the IMDC classification and several nutritional and inflammatory markers: PNI (*r* =  – 0.48), GNRI (*r* =  – 0.51), GPS (*r* = 0.49), and SII (*r* = 0.46). By contrast, BMI showed moderate to strong correlations with body composition and nutritional indices, including SMI (*r* = 0.63), VATI (*r* = 0.74), SATI (*r* = 0.75), PNI (*r* = 0.38), and GNRI (*r* = 0.53). The adipose tissue-related indices VATI and SATI demonstrated moderate correlations with the nutritional marker GNRI (*r* = 0.44 and 0.42, respectively). Notably, both PNI and GNRI demonstrated strong correlations with each other (*r* = 0.92) and moderate associations with inflammatory markers, body composition indices, and IMDC classification, highlighting their roles as well-balanced and comprehensive prognostic markers.

## Discussion

The relationship between obesity and survival in patients with mRCC has been widely reported, but varying results have been obtained depending on the obesity index used. While BMI has been identified as an independent prognostic factor in several studies [5, 21, 22], others have shown that visceral or subcutaneous adipose tissue, rather than BMI, more accurately predict survival outcomes [23, 24]. CT imaging enables separate measurements of visceral and subcutaneous adipose tissue, offering more precise body composition data than BMI. In our study, both VATI and SATI demonstrated stronger associations with prolonged survival compared with BMI, which may be partly explained by the lower prevalence of overweight (BMI ≥ 25 kg/m^2^) in the Japanese population, and the fact that BMI does not directly reflect adipose tissue distribution [25].

Although obesity is a recognized risk factor for RCC incidence [3], paradoxically, several studies have reported a favorable impact of obesity on OS in advanced RCC [4, 5]. Proposed explanations include the less aggressive nature of RCC in obese patients and lead-time bias because of cancer cachexia. Higher BMI has been associated with more favorable clinicopathologic features at diagnosis, such as lower stage, lower Fuhrman grade, and smaller tumor size [26]. Our findings demonstrate that adipose tissue indices are moderately correlated with nutritional markers but show weaker correlations with the IMDC classification (Fig. 2), suggesting that while adipose tissue-related indices partially reflect nutritional status, they may have distinct prognostic significance independent of conventional risk models. Furthermore, IL-6, which is preferentially released from visceral rather than subcutaneous adipose tissue in obese individuals [27], has been reported to attenuate the efficacy of ICIs [28]. By contrast, adiponectin, an anti-inflammatory adipokine secreted abundantly from subcutaneous rather than visceral adipose tissue, [29] has been suggested to suppress immune-related adverse events and potentially enhance treatment continuity [30]. Separately, obesity-associated hyperleptinemia is reported to induce PD-1 expression on T cells and macrophages, thereby enhancing sensitivity to ICIs [31]. The role of subcutaneous adipose tissue further extends to functioning as a vast reservoir for memory T cells, which mediate anti-tumor responses, and it is reported to metabolically support their long-term survival and functional maintenance through fatty acid oxidation [32]. The synergistic effects of these factors are believed to collectively contribute to prolonged survival. Consistent with these observations, our findings suggest that the association between VATI and OS was attenuated in the ICI-based regimen group; however, the role of visceral adipose tissue in modulating ICI efficacy remains unclear and warrants further investigation.

This study used PNI and GNRI as nutritional indices, and GPS was applied as a composite index of nutrition and systemic inflammation. Consistent with previous reports [8, 13, 33], these indices were significantly associated with OS in patients with mRCC, and they were strongly correlated with each other, possibly because of the influence of albumin as a common factor. A favorable nutritional status could enhance immune competence, attenuate systemic inflammation, preserve metabolic reserves, and improve treatment tolerance, all of which may collectively contribute to improved survival outcomes in cancer patients. Meanwhile, malnutrition is known to adversely affect the immune system through several mechanisms, including the induction and exacerbation of systemic chronic inflammation [34], impairment of T-cell production and function [35], induction of an immunosuppressive state via cachexia [36], and dysbiosis of the gut microbiota [37]. Collectively, these factors may contribute to resistance to ICI therapy. In the ICI treatment group, these indices remained strong predictors of OS, indicating that poor nutritional status continues to be an adverse prognostic factor even in the era of ICI therapy. Although such associations have been reported in other cancer types [38], to our knowledge, no previous study has specifically examined the association between PNI, GNRI, GPS, and outcomes in mRCC patients treated with ICIs, highlighting the novelty and clinical relevance of our findings.

Systemic inflammatory markers have been reported to be associated with survival in patients with mRCC in several meta-analyses [39–41]. In particular, the prognostic and predictive significance of SII [20, 42], NLR [42], PLR [42], and LMR [43] have been suggested in small cohort studies of patients with mRCC treated with first-line ICI-based regimens, especially the combination of ipilimumab and nivolumab. A recent study of 112 patients with mRCC who received ipilimumab and nivolumab combination therapy reported that lower LMR was most strongly associated with shorter OS [43]. In the present study, LMR also exhibited the highest prognostic performance among inflammatory markers in ICI-based regimens, and these findings were consistent with reports in other cancer types. Although the biological significance of LMR on ICI-based treatment remains unclear, tumor-associated macrophages (TAMs), especially pro-tumorigenic M2-type macrophages, are known to contribute to tumor progression by promoting immunosuppression, angiogenesis, and cancer cell proliferation and invasion [44, 45] M2 macrophages are suggested to suppress the anti-tumor function of T cells through PD-L1 expression, secretion of immunosuppressive cytokines, and induction of regulatory T cells, implying that higher infiltration of M2 macrophages is associated with resistance to ICI treatment and poorer prognosis [46, 47]. Circulating monocyte levels have been reported to be associated with the presence of TAMs [48], suggesting that a low LMR may serve as a biomarker for ICI resistance.

The predictive accuracy of these markers has varied across studies, and few investigations have directly compared their prognostic value between the non-ICI-based regimen era and the ICI-based regimen era. In the subgroup analysis of the present study, while some markers lost significant associations with OS, the prognostic utility of markers identified during the non-ICI-based regimen era was largely preserved in the ICI-based regimen era. Despite a considerable improvement in the prognosis of mRCC in the ICI-based regimen era compared to previous periods (median NR vs. 22.2 months), the survival for patients in each poor prognostic group showed minimal prolongation for most of the markers (Fig. 1). This study is thus considered to provide foundational data for developing new risk stratification schemes in the ICI-treated patients, aimed at identifying patients with poor prognosis and targeting them for future therapeutic strategies. These findings further underscore the need for standardized definitions and large-scale validation in diverse patient populations. To establish more accurate and clinically applicable risk stratification models, it will be essential to incorporate these inflammatory markers into predictive algorithms validated through prospective studies. Importantly, such efforts will require the adoption of fixed, standardized cut-off values to ensure consistency and comparability across future trials.

This study has several limitations. First, among the 136 patients with mRCC who received first-line systemic therapy, only 52 patients were treated with regimens including ICIs. Therefore, the sample size might have been insufficient to adequately evaluate the impact of each index on OS in this subgroup. Second, the study could not examine the association between the indices and the treatment response or progression-free survival as an endpoint. An accurate evaluation of these endpoints was difficult and was not included because the study used retrospective real-world data. Third, the lack of universally established cut-off values represents a significant limitation, not only for body composition indices but also for other indices with continuous values. For systemic inflammation indices, cut-off values that are generally considered acceptable were employed; therefore, the C-index for each index may not correspond directly to its HR value. While continuous variables were dichotomized for analytical purposes, it is conceivable that categorization into three or four groups would yield greater clinical utility, in which case the C-index values would also be expected to differ.

In conclusion, several indices reflecting body composition, nutritional status, and systemic inflammation remain valuable prognostic markers in patients with mRCC receiving ICI-based first-line therapy. In particular, SATI and PNI demonstrated strong predictive performance, allowing clear stratification of OS. These findings suggest that incorporating such markers into current prognostic models may improve risk stratification and guide treatment decisions in the era of ICI-based first-line regimens.

## Supplementary Information

Below is the link to the electronic supplementary material.

Supplementary file1 (PPTX 36 KB)

Supplementary file2 (DOCX 23 KB)

Supplementary file3 (DOCX 21 KB)

Supplementary file4 (DOCX 21 KB)

## Data Availability

The datasets generated and analyzed in the current study are available from the corresponding author upon reasonable request.

## References

[1] Naito S, Kato T, Numakura K et al (2021) Prognosis of Japanese metastatic renal cell carcinoma patients in the targeted therapy era. Int J Clin Oncol 26:1947–1954. 10.1007/s10147-021-01979-9 34191191 10.1007/s10147-021-01979-9

[2] Heng DY, Xie W, Regan MM et al (2013) External validation and comparison with other models of the International Metastatic Renal-Cell Carcinoma Database Consortium prognostic model: a population-based study. Lancet Oncol 14:141–148. 10.1016/S1470-2045(12)70559-4 23312463 10.1016/S1470-2045(12)70559-4 PMC4144042

[3] Renehan AG, Tyson M, Egger M et al (2008) Body-mass index and incidence of cancer: a systematic review and meta-analysis of prospective observational studies. Lancet 371:569–578. 10.1016/S0140-6736(08)60269-X 18280327 10.1016/S0140-6736(08)60269-X

[4] Turco F, Tucci M, Di Stefano RF et al (2021) Renal cell carcinoma (RCC): fatter is better? A review on the role of obesity in RCC. Endocr Relat Cancer 28:R207–R216. 10.1530/ERC-20-0457 33949971 10.1530/ERC-20-0457

[5] Ged Y, Sanchez A, Patil S et al (2022) Associations between pretreatment body composition features and clinical outcomes among patients with metastatic clear cell renal cell carcinoma treated with immune checkpoint blockade. Clin Cancer Res 28:5180–5189. 10.1158/1078-0432.CCR-22-1389 36190538 10.1158/1078-0432.CCR-22-1389 PMC9793646

[6] Lee DH, Giovannucci EL (2019) The obesity paradox in cancer: epidemiologic insights and perspectives. Curr Nutr Rep 8:175–181. 10.1007/s13668-019-00280-6 31129887 10.1007/s13668-019-00280-6

[7] Elagizi A, Kachur S, Lavie CJ et al (2018) An overview and update on obesity and the obesity paradox in cardiovascular diseases. Prog Cardiovasc Dis 61:142–150. 10.1016/j.pcad.2018.07.003 29981771 10.1016/j.pcad.2018.07.003

[8] Xiong S-C, Hu X, Lia T et al (2022) Prognostic significance of prognostic nutritional index in patients with renal cell carcinoma: a meta-analysis. Nutr Cancer 74:860–868. 10.1080/01635581.2021.1931702 34060398 10.1080/01635581.2021.1931702

[9] Teishima J, Inoue S, Hayashi T et al (2019) Current status of prognostic factors in patients with metastatic renal cell carcinoma. Int J Urol 26:608–617. 10.1111/iju.13956 30959579 10.1111/iju.13956

[10] Onodera T, Goseki N, Kosaki G (1984) Prognostic nutritional index in gastrointestinal surgery of malnourished cancer patients. Nihon Geka Gakkai Zasshi 85:1001–1005 6438478

[11] Bouillanne O, Morineau G, Dupont C et al (2005) Geriatric nutritional risk index: a new index for evaluating at-risk elderly medical patients. Am J Clin Nutr 82:777–783. 10.1093/ajcn/82.4.777 16210706 10.1093/ajcn/82.4.777

[12] Semeniuk-Wojtaś A, Lubas A, Stec R et al (2018) Neutrophil-to-lymphocyte ratio, platelet-to-lymphocyte ratio, and C-reactive protein as new and simple prognostic factors in patients with metastatic renal cell cancer treated with tyrosine kinase inhibitors: a systemic review and meta-analysis. Clin Genitourin Cancer 16:e685–e693. 10.1016/j.clgc.2018.01.010 29454639 10.1016/j.clgc.2018.01.010

[13] Tong T, Guan Y, Xiong H et al (2020) A meta-analysis of Glasgow prognostic score and modified Glasgow prognostic score as biomarkers for predicting survival outcome in renal cell carcinoma. Front Oncol 10:1541. 10.3389/fonc.2020.01541 33042799 10.3389/fonc.2020.01541 PMC7527435

[14] Tang Y, Liang J, Liu Z et al (2021) Clinical significance of prognostic nutritional index in renal cell carcinomas. Medicine (Baltimore) 100:e25127. 10.1097/MD.0000000000025127 33725913 10.1097/MD.0000000000025127 PMC7969234

[15] Paris MT, Tandon P, Heyland DK et al (2020) Automated body composition analysis of clinically acquired computed tomography scans using neural networks. Clin Nutr 39:3049–3055. 10.1016/j.clnu.2020.01.008 32007318 10.1016/j.clnu.2020.01.008 PMC7374050

[16] Martin L, Birdsell L, MacDonald N et al (2013) Cancer cachexia in the age of obesity: skeletal muscle depletion is a powerful prognostic factor, independent of body mass index. J Clin Oncol 31:1539–1547. 10.1200/jco.2012.45.2722 23530101 10.1200/JCO.2012.45.2722

[17] Zhang X, Zhang J, Liu F et al (2023) Prognostic nutritional index (PNI) as a predictor in patients with metabolic syndrome and heart failure. Diabetes Metab Syndr Obes 16:2503–2514. 10.2147/DMSO.S420924 37614379 10.2147/DMSO.S420924 PMC10443633

[18] Zahorec R (2001) Ratio of neutrophil to lymphocyte counts–rapid and simple parameter of systemic inflammation and stress in critically ill. Bratisl Lek Listy 102:5–14 11723675

[19] Hu B, Yang X-R, Xu Y et al (2014) Systemic immune-inflammation index predicts prognosis of patients after curative resection for hepatocellular carcinoma. Clin Cancer Res 20:6212–6222. 10.1158/1078-0432.CCR-14-0442 25271081 10.1158/1078-0432.CCR-14-0442

[20] Stühler V, Herrmann L, Rausch S et al (2022) Role of the systemic immune-inflammation index in patients with metastatic renal cell carcinoma treated with first-line ipilimumab plus nivolumab. Cancers (Basel) 14:2972. 10.3390/cancers14122972 35740636 10.3390/cancers14122972 PMC9221331

[21] Sanchez A, Furberg H, Kuo F et al (2020) Transcriptomic signatures related to the obesity paradox in patients with clear cell renal cell carcinoma: a cohort study. Lancet Oncol 21:283–293. 10.1016/S1470-2045(19)30797-1 31870811 10.1016/S1470-2045(19)30797-1 PMC7082892

[22] Albiges L, Hakimi AA, Xie W et al (2016) Body mass index and metastatic renal cell carcinoma: clinical and biological correlations. J Clin Oncol 34:3655–3663. 10.1200/JCO.2016.66.7311 27601543 10.1200/JCO.2016.66.7311 PMC5065111

[23] Mizuno R, Miyajima A, Hibi T et al (2017) Impact of baseline visceral fat accumulation on prognosis in patients with metastatic renal cell carcinoma treated with systemic therapy. Med Oncol 34:47. 10.1007/s12032-017-0908-3 28213730 10.1007/s12032-017-0908-3

[24] Gu W, Zhu Y, Wang H et al (2015) Prognostic value of components of body composition in patients treated with targeted therapy for advanced renal cell carcinoma: a retrospective case series. PLoS One 10:e0118022. 10.1371/journal.pone.0118022 25668688 10.1371/journal.pone.0118022 PMC4323238

[25] Chatterjee S, Kleinman N, Gharajeh A et al (2009) Computerized tomography measurement of visceral adiposity predicts plasma adiponectin levels and metastatic disease in patients with clear cell renal cell carcinoma. Curr Urol 2:188–193. 10.1159/000209831

[26] Choi Y, Park B, Jeong BC et al (2013) Body mass index and survival in patients with renal cell carcinoma: a clinical-based cohort and meta-analysis. Int J Cancer 132:625–634. 10.1002/ijc.27639 22610826 10.1002/ijc.27639

[27] Wueest S, Konrad D (2020) The controversial role of IL-6 in adipose tissue on obesity-induced dysregulation of glucose metabolism. Am J Physiol-Endocrinol Metab 319:E607–E613. 10.1152/ajpendo.00306.2020 32715746 10.1152/ajpendo.00306.2020

[28] Laino AS, Woods D, Vassallo M et al (2020) Serum interleukin-6 and C-reactive protein are associated with survival in melanoma patients receiving immune checkpoint inhibition. J Immunother Cancer 8:e000842. 10.1136/jitc-2020-000842 32581042 10.1136/jitc-2020-000842 PMC7312339

[29] Frederiksen L, Nielsen TL, Wraae K et al (2009) Subcutaneous rather than visceral adipose tissue is associated with adiponectin levels and insulin resistance in young men. J Clin Endocrinol Metab 94:4010–4015. 10.1210/jc.2009-0980 19755479 10.1210/jc.2009-0980

[30] Han S-J, Glatman Zaretsky A, Andrade-Oliveira V et al (2017) White adipose tissue is a reservoir for memory T cells and promotes protective memory responses to infection. Immunity 47:1154-1168.e6. 10.1016/j.immuni.2017.11.009 29221731 10.1016/j.immuni.2017.11.009 PMC5773068

[31] Wang Z, Aguilar EG, Luna JI et al (2019) Paradoxical effects of obesity on T cell function during tumor progression and PD-1 checkpoint blockade. Nat Med 25:141–151. 10.1038/s41591-018-0221-5 30420753 10.1038/s41591-018-0221-5 PMC6324991

[32] Mastrolonardo EV, Llerena P, De Ravin E et al (2025) Improved survival with elevated BMI following immune checkpoint inhibition across various solid tumor cancer types. Cancer 131:e35799. 10.1002/cncr.35799 40069917 10.1002/cncr.35799 PMC11897419

[33] Saal J, Bald T, Eckstein M et al (2023) Integrating on-treatment modified Glasgow prognostic score and imaging to predict response and outcomes in metastatic renal cell carcinoma. JAMA Oncol 9:1048. 10.1001/jamaoncol.2023.1822 37347489 10.1001/jamaoncol.2023.1822 PMC10288377

[34] Ye H, Li M (2025) Baseline (modified) Glasgow prognostic score as a predictor of therapeutic response to immune checkpoint inhibitors in solid tumors: a systematic review and meta-analysis. Oncol Lett. 10.3892/ol.2025.14931 40007624 10.3892/ol.2025.14931 PMC11851447

[35] Rangel Rivera GO, Knochelmann HM, Dwyer CJ et al (2021) Fundamentals of T cell metabolism and strategies to enhance cancer immunotherapy. Front Immunol. 10.3389/fimmu.2021.645242 33815400 10.3389/fimmu.2021.645242 PMC8014042

[36] Rounis K, Makrakis D, Tsigkas A-P et al (2021) Cancer cachexia syndrome and clinical outcome in patients with metastatic non-small cell lung cancer treated with PD-1/PD-L1 inhibitors: results from a prospective, observational study. Transl Lung Cancer Res 10:3538–3549. 10.21037/tlcr-21-460 34584855 10.21037/tlcr-21-460 PMC8435387

[37] Raoul P, De Gaetano V, Sciaraffia G et al (2024) Gastric cancer, immunotherapy, and nutrition: the role of microbiota. Pathog Basel Switz 13:357. 10.3390/pathogens13050357 10.3390/pathogens13050357 PMC11124250 38787209

[38] Ni L, Huang J, Ding J et al (2022) Prognostic nutritional index predicts response and prognosis in cancer patients treated with immune checkpoint inhibitors: a systematic review and meta-analysis. Front Nutr 9:823087. 10.3389/fnut.2022.823087 35938131 10.3389/fnut.2022.823087 PMC9353139

[39] Shao Y, Wu B, Jia W et al (2020) Prognostic value of pretreatment neutrophil-to-lymphocyte ratio in renal cell carcinoma: a systematic review and meta-analysis. BMC Urol 20:90. 10.1186/s12894-020-00665-8 32631294 10.1186/s12894-020-00665-8 PMC7339475

[40] Jin M, Yuan S, Yuan Y et al (2021) Prognostic and clinicopathological significance of the systemic immune-inflammation index in patients with renal cell carcinoma: a meta-analysis. Front Oncol 11:735803. 10.3389/fonc.2021.735803 34950577 10.3389/fonc.2021.735803 PMC8689141

[41] Chen X, Meng F, Jiang R (2021) Neutrophil-to-lymphocyte ratio as a prognostic biomarker for patients with metastatic renal cell carcinoma treated with immune checkpoint inhibitors: a systematic review and meta-analysis. Front Oncol 11:746976. 10.3389/fonc.2021.746976 34900692 10.3389/fonc.2021.746976 PMC8660071

[42] Iinuma K, Enomoto T, Kawada K et al (2021) Utility of neutrophil-to-lymphocyte ratio, platelet-to-lymphocyte ratio, and systemic immune inflammation index as prognostic, predictive biomarkers in patients with metastatic renal cell carcinoma treated with Nivolumab and Ipilimumab. J Clin Med 10:5325. 10.3390/jcm10225325 34830607 10.3390/jcm10225325 PMC8617687

[43] Numakura K, Sekine Y, Osawa T et al (2024) The lymphocyte-to-monocyte ratio as a significant inflammatory marker associated with survival of patients with metastatic renal cell carcinoma treated using nivolumab plus ipilimumab therapy. Int J Clin Oncol 29:1019–1026. 10.1007/s10147-024-02538-8 38797782 10.1007/s10147-024-02538-8

[44] Van Dalen FJ, Van Stevendaal MHME, Fennemann FL et al (2018) Molecular repolarisation of tumour-associated macrophages. Molecules 24:9. 10.3390/molecules24010009 30577495 10.3390/molecules24010009 PMC6337345

[45] Lin Y, Xu J, Lan H (2019) Tumor-associated macrophages in tumor metastasis: biological roles and clinical therapeutic applications. J Hematol OncolJ Hematol Oncol 12:76. 10.1186/s13045-019-0760-3 31300030 10.1186/s13045-019-0760-3 PMC6626377

[46] Chen Y, Tan W, Wang C (2018) Tumor-associated macrophage-derived cytokines enhance cancer stem-like characteristics through epithelial-mesenchymal transition. OncoTargets Ther 11:3817–3826. 10.2147/OTT.S168317 10.2147/OTT.S168317 PMC6038883 30013362

[47] Kashima S, Rout R, Hugaboom M et al (2025) Investigation of tumor-associated macrophages (TAMs) and therapeutic resistance to immune checkpoint inhibitors (ICI) through single-cell analysis of renal cell carcinoma (RCC). J Clin Oncol 43:4527–4527. 10.1200/jco.2025.43.16_suppl.4527

[48] Yuan J, Zhao X, Li Y et al (2022) The association between blood indexes and immune cell concentrations in the primary tumor microenvironment predicting survival of immunotherapy in gastric cancer. Cancers 14:3608. 10.3390/cancers14153608 35892867 10.3390/cancers14153608 PMC9332606

