# Supplementary material for: Reassessing prognostic markers in metastatic renal cell carcinoma in the era of immune checkpoint inhibitors: the enduring value of body composition, nutritional, and inflammatory indices
Source: Int J Clin Oncol. 2026 Jan 23;31(3):418–27. doi: 10.1007/s10147-025-02855-6 (PMC12932366; doi:10.1007/s10147-025-02855-6)
Supplement: Supplementary file 1 — Supplementary file1 (PPTX 36 KB) [file 10147_2025_2855_MOESM1_ESM.pptx]

## Slide 1
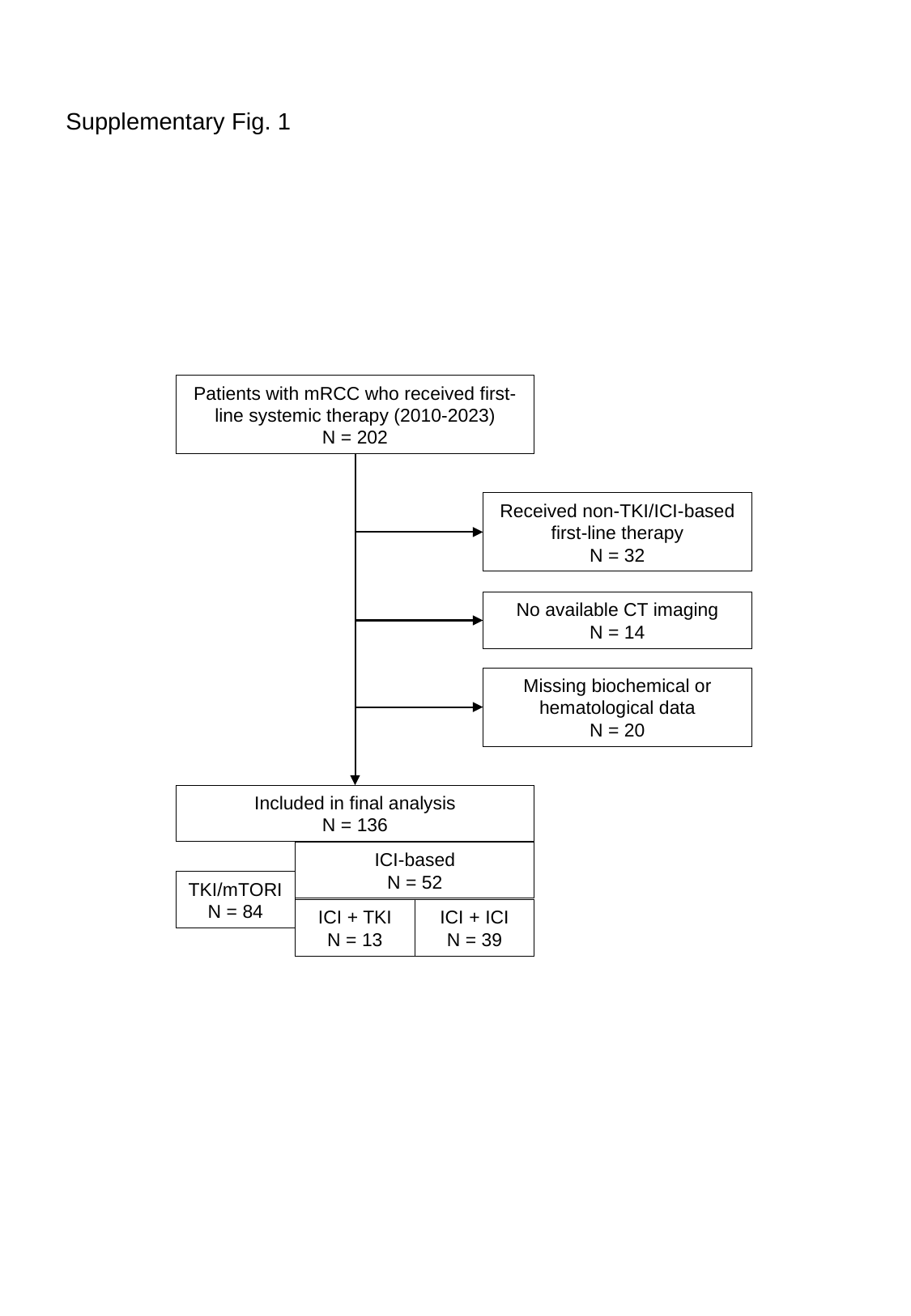

Supplementary Fig. 1
Patients with mRCC who received first-line systemic therapy (2010-2023)
N = 202
Received non-TKI/ICI-based first-line therapy
N = 32
No available CT imaging
N = 14
Missing biochemical or hematological data
N = 20
Included in final analysis
N = 136
ICI-based
N = 52
TKI/mTORI
N = 84
ICI + ICI
N = 39
ICI + TKI
N = 13
