# Supplementary material for: Reassessing prognostic markers in metastatic renal cell carcinoma in the era of immune checkpoint inhibitors: the enduring value of body composition, nutritional, and inflammatory indices
Source: Int J Clin Oncol. 2026 Jan 23;31(3):418–27. doi: 10.1007/s10147-025-02855-6 (PMC12932366; doi:10.1007/s10147-025-02855-6)
Supplement: Supplementary file 2 — Supplementary file2 (DOCX 23 KB) [file 10147_2025_2855_MOESM2_ESM.docx]

| Variable | Overall (n = 136) | Male (n = 108) | Female (n = 28) | *P* |  |
| --- | --- | --- | --- | --- | --- |
| Age, year (IQR) | 66 (61, 71) | 66 (61, 71) | 67 (61.5, 73) | 0.661 |  |
| IMDC Favorable (%) | 12 (8.8) | 9 (8.3) | 3 (10.7) | 0.125 |  |
| Intermediate (%) | 71 (52.2) | 61 (56.5) | 10 (35.7) |  |  |
| Poor (%) | 53 (39.0) | 38 (35.2) | 15 (53.6) |  |  |
| Body height, cm (IQR) | 163.5 (158.0, 167.2) | 164.6 (161.1, 169.2) | 153.7 (150.0, 157.1) | <0.001 |  |
| Body weight, kg (IQR) | 60.7 (51.2, 66.4) | 62.6 (55.0, 68.1) | 49.8 (41.0, 54.0) | <0.001 |  |
| BMI, kg/m² (IQR) | 22.5 (20.2, 24.5) | 22.7 (20.8, 24.7) | 20.7 (18.0, 23.5) | 0.009 |  |
| SMI, cm²/m² (IQR) | 46.1 (41.4, 52.1) | 48.7 (45.1, 54.5) | 37.1 (33.2, 41.1) | <0.001 |  |
| VATI, cm²/m² (IQR) | 27.8 (11.4, 48.6) | 33.4 (14.5, 50.9) | 12.4 (2.8, 27.1) | <0.001 |  |
| SATI, cm²/m² (IQR) | 32.6 (21.2, 45.0) | 32.2 (23.7, 43.4) | 35.5 (14.9, 65.5) | 0.761 |  |
| VSR (IQR) | 0.84 (0.49, 1.22) | 0.9 (0.6, 1.3) | 0.4 (0.3, 0.6) | <0.001 |  |
| PNI (IQR) | 43.3 (36.5, 48.7) | 43.5 (36.7, 48.8) | 42.4 (33.9, 47.5) | 0.236 |  |
| GNRI (IQR) | 93.8 (86.4, 101.3) | 95.8 (87.1, 101.6) | 90.8 (78.6, 99.4) | 0.067 |  |
| GPS 0 (%) | | 63 (46.3) | 54 (50.0) | 9 (32.1) | 0.184 |
| 1 (%) | | 24 (17.6) | 17 (15.7) | 7 (25.0) |  |
| 2 (%) | | 49 (36.0) | 37 (34.3) | 12 (42.9) |  |
| SII, x10^9^/L (IQR) | 874.0 (531.8, 1333.3) | 817.7 (531.8, 1299.0) | 981.9 (542.5, 1684.9) | 0.201 |  |
| NLR (IQR) | 3.3 (2.2, 4.5) | 3.2 (2.4, 4.5) | 3.4 (2.1, 4.7) | 0.914 |  |
| PLR (IQR) | 215.5 (142.5, 283.9) | 206.4 (130.3, 256.4) | 261.5 (154.6, 331.9) | 0.031 |  |
| LMR (IQR) | 3.2 (2.4, 4.7) | 3.2 (2.4, 4.6) | 3.4 (2.2, 4.8) | 0.712 |  |
| ICI, immune checkpoint inhibitor; IMDC, International mRCC Database Consortium; BMI, body mass index; SMI, skeletal muscle index; VATI, visceral adipose tissue index; SATI, subcutaneous adipose tissue index; VSR, visceral to subcutaneous adipose tissue ratio; PNI, prognostic nutritional index; GNRI, geriatric nutritional risk index; GPS, Glasgow prognostic score; SII, systemic immune-inflammation index; NLR, neutrophil to lymphocyte ratio; PLR, platelet to lymphocyte ratio; LMR, lymphocyte to monocyte ratio | | | | |  |

Supplementary Table S1 Comparison of body composition, nutritional, and systemic inflammatory indices between male and female patients
