# Supplementary material for: Reassessing prognostic markers in metastatic renal cell carcinoma in the era of immune checkpoint inhibitors: the enduring value of body composition, nutritional, and inflammatory indices
Source: Int J Clin Oncol. 2026 Jan 23;31(3):418–27. doi: 10.1007/s10147-025-02855-6 (PMC12932366; doi:10.1007/s10147-025-02855-6)
Supplement: Supplementary file 3 — Supplementary file3 (DOCX 21 KB) [file 10147_2025_2855_MOESM3_ESM.docx]

Supplementary Table S2 Comparison of body composition, nutritional, and systemic inflammatory indices between non-ICI and ICI-based regimen groups

| Variable | Overall (n = 136) | Non-ICI-based regimen (n = 84) | ICI-based regimen (n = 52) | *P* |  |
| --- | --- | --- | --- | --- | --- |
| Age, year (IQR) | 66 (61, 71) | 65.5 (61, 70.5) | 68 (63.5, 71.5) | 0.165 |  |
| IMDC Favorable (%) | 12 (8.8) | 7 (8.3) | 5 (9.6) | 0.164 |  |
| Intermediate (%) | 72 (52.6) | 49 (58.3) | 22 (42.3) |  |  |
| Poor (%) | 53 (38.7) | 28 (33.3) | 25 (48.1) |  |  |
| Body height, cm (IQR) | 163.5 (158.0, 167.4) | 163.5 (157.0, 166.8) | 163.6 (158.3, 169.1) | 0.662 |  |
| Body weight, kg (IQR) | 60.7 (51.4, 66.5) | 60.7 (50.9, 65.6) | 60.4 (51.9, 70.2) | 0.465 |  |
| BMI, kg/m² (IQR) | 22.5 (20.2, 24.5) | 22.7 (20.4, 24.6) | 22.0 (19.9, 24.2) | 0.895 |  |
| SMI, cm²/m² (IQR) | 46.2 (41.6, 52.3) | 45.6 (41.4, 51.9) | 46.4 (41.1, 53.7) | 0.630 |  |
| VATI, cm²/m² (IQR) | 28.0 (11.4, 48.6) | 32.9 (12.9, 48.6) | 22.2 (10.6, 44.6) | 0.585 |  |
| SATI, cm²/m² (IQR) | 32.8 (21.6, 45.5) | 32.8 (22.9, 44.2) | 32.1 (20.7, 48.1) | 0.986 |  |
| VSR (IQR) | 0.8 (0.5, 1.2) | 0.9 (0.5, 1.2) | 0.8 (0.5, 1.2) | 0.380 |  |
| PNI (IQR) | 43.3 (36.6, 48.7) | 43.8 (36.9, 48.8) | 41.1 (36.2, 48.4) | 0.184 |  |
| GNRI (IQR) | 93.8 (86.4, 101.3) | 96.8 (86.4, 101.6) | 90.8 (85.2, 101.3) | 0.274 |  |
| GPS 0 (%) | 63 (46.0) | 40 (47.6) | 23 (44.2) | 0.180 | |
| 1 (%) | 25 (18.2) | 18 (21.4) | 6 (11.5) |  | |
| 2 (%) | 49 (35.8) | 26 (31.0) | 23 (44.2) |  | |
| SII, x10^9^/L (IQR) | 868.5 (536.8, 1319.2) | 814.7 (487.1, 1147.4) | 1028.1 (584.7, 1537.6) | 0.078 |  |
| NLR (IQR) | 3.3 (2.2, 4.5) | 3.3 (2.2, 4.5) | 3.3 (2.5, 4.8) | 0.651 |  |
| PLR (IQR) | 215.3 (144.1, 283.3) | 205.6 (127.6, 258.3) | 232.5 (158.7, 308.9) | 0.050 |  |
| LMR (IQR) | 3.2 (2.4, 4.7) | 3.2 (2.4, 4.7) | 3.2 (2.4, 4.7) | 0.710 |  |

ICI, immune checkpoint inhibitor; IMDC, International mRCC Database Consortium; BMI, body mass index; SMI, skeletal muscle index; VATI, visceral adipose tissue index; SATI, subcutaneous adipose tissue index; VSR, visceral to subcutaneous adipose tissue ratio; PNI, prognostic nutritional index; GNRI, geriatric nutritional risk index; GPS, Glasgow prognostic score; SII, systemic immune-inflammation index; NLR, neutrophil to lymphocyte ratio; PLR, platelet to lymphocyte ratio; LMR, lymphocyte to monocyte ratio
