# Supplementary material for: Reassessing prognostic markers in metastatic renal cell carcinoma in the era of immune checkpoint inhibitors: the enduring value of body composition, nutritional, and inflammatory indices
Source: Int J Clin Oncol. 2026 Jan 23;31(3):418–27. doi: 10.1007/s10147-025-02855-6 (PMC12932366; doi:10.1007/s10147-025-02855-6)
Supplement: Supplementary file 4 — Supplementary file4 (DOCX 21 KB) [file 10147_2025_2855_MOESM4_ESM.docx]

**Supplementary Table S3** Prognostic performance (Harrell's C-index) of body composition, nutritional, and inflammatory indices for overall survival

| Index | Overall | Non-ICI-based regimen | ICI-based regimen |
| --- | --- | --- | --- |
| IMDC (Ordinal) | 0.638 | 0.644 | 0.667 |
| BMI (Continuous) | 0.572 | 0.588 | 0.571 |
| (Ordinal) | 0.565 | 0.576 | 0.537 |
| SMI (Continuous) | 0.560 | 0.561 | 0.561 |
| (Ordinal, [Sarcopenia]) | 0.533 | 0.501 | 0.604 |
| VATI (Continuous) | 0.595 | 0.616 | 0.599 |
| (Ordinal) | 0.592 | 0.597 | 0.606 |
| SATI (Continuous) | 0.612 | 0.588 | 0.679 |
| (Ordinal) | 0.608 | 0.576 | 0.690 |
| VSR (Continuous) | 0.541 | 0.578 | 0.512 |
| (Ordinal) | 0.503 | 0.522 | 0.534 |
| PNI (Continuous) | 0.688 | 0.684 | 0.759 |
| (Ordinal) | 0.659 | 0.647 | 0.736 |
| GNRI (Continuous) | 0.692 | 0.679 | 0.773 |
| (Ordinal) | 0.669 | 0.661 | 0.730 |
| GPS (Ordinal) | 0.668 | 0.663 | 0.708 |
| SII (Continuous) | 0.628 | 0.652 | 0.610 |
| (Ordinal) | 0.602 | 0.602 | 0.619 |
| NLR (Continuous) | 0.626 | 0.643 | 0.620 |
| (Ordinal) | 0.599 | 0.593 | 0.633 |
| PLR (Continuous) | 0.605 | 0.649 | 0.575 |
| (Ordinal) | 0.579 | 0.595 | 0.579 |
| LMR (Continuous) | 0.637 | 0.643 | 0.661 |
| (Ordinal) | 0.595 | 0.572 | 0.662 |

ICI, immune checkpoint inhibitor; IMDC, International mRCC Database Consortium; BMI, body mass index; SMI, skeletal muscle index; VATI, visceral adipose tissue index; SATI, subcutaneous adipose tissue index; VSR, visceral to subcutaneous adipose tissue ratio; PNI, prognostic nutritional index; GNRI, geriatric nutritional risk index; GPS, Glasgow prognostic score; SII, systemic immune-inflammation index; NLR, neutrophil to lymphocyte ratio; PLR, platelet to lymphocyte ratio; LMR, lymphocyte to monocyte ratio
